# Diabetes mellitus and the risk of multidrug resistant tuberculosis: a meta-analysis

**DOI:** 10.1038/s41598-017-01213-5

**Published:** 2017-04-24

**Authors:** Qianqian Liu, Wenzhang Li, Miao Xue, Yunfeng Chen, Xinmiao Du, Chengdi Wang, Lina Han, Yin Tang, Yulin Feng, Chuanmin Tao, Jian-Qing He

**Affiliations:** 1 Department of Respiratory Diseases, Chengdu Municipal First People’s Hospital, Chengdu, Sichuan 610041 China; 2 Department of Respiratory and Critical Care Medicine, West China Hospital, Sichuan University, Chengdu, Sichuan 610041 China; 3 Department of Cardiology, First Affiliated Hospital of Chengdu Medical College, Chengdu, Sichuan 610500 China; 4 Department of Endocrinology, West China Hospital, Sichuan University, Chengdu, Sichuan 610041 China; 5 State Key Laboratory of Oral Disease, West China School & Hospital of Stomotology, Sichuan University, Chengdu, Sichuan 610041 China; 6 Department of Laboratory Medicine, West China Hospital, Sichuan University, Chengdu, Sichuan 610041 China

## Abstract

The high prevalence of diabetes mellitus (DM) among multidrug resistant tuberculosis (MDR-TB) patients is a serious cause for concern. We conducted a meta-analysis to determine whether DM is an independent risk factor for MDR-TB. Electronic literature searches of the PubMed, Web of Science and EMBASE databases up to July 12, 2016 were conducted. The pooled adjusted odds ratio (OR) and 95% confidence intervals (CIs) were calculated using the random effects model with STATA 12.0 software. In total 13 studies, including 9289 individuals with TB, were included in this meta-analysis. Significant association between DM and MDR-TB (OR = 1.71; 95% CI = 1.32, 2.22) was identified. Subgroup analyses showed that: 1) Pooled OR was 1.25 (95% CI: 0.82–1.91) for cross-sectional studies, and was 2.14 (95% CI: 1.51–3.02) for longitudinal studies; 2) The pooled OR was 1.69 (95% CI:1.09–2.62) for primary MDR-TB, 1.94 (95% CI:1.42–2.65) for any MDR-TB, and 0.85 for secondary MDR-TB (95% CI: 0.29–2.54); 3) DM was significantly associated with MDR-TB in both Caucasian (OR = 2.26, 95% CI: 1.66–3.07) and Asian (OR = 1.40, 95% CI: 1.01–1.95) subgroups. No evidence of publication bias was identified. In conclusion, the pooling analysis indicated that DM was an independent risk factor for MDR-TB, especially for primary MDR-TB.

## Introduction

The emergence of MDR-TB (multidrug resistant tuberculosis), caused by *M*. *tuberculosis* (MTB) that is resistant to at least isoniazid and rifampicin, is posing a great threat to global public health. Despite effective preventive and therapeutic methods that have been actively promoted worldwide, it is still estimated that 3.3% of newly diagnosed tuberculosis (TB) cases and 20% of previously treated TB cases have MDR-TB according to WHO reports^1^. The treatment of patients infected with MDR-TB strains is extremely challenging due to the complexity of chemotherapy regimens and the toxicity of alternative drugs. Furthermore, treatment of MDR-TB imposes a huge financial burden on public health systems. However, compared with the cure rate of 96% in drug-susceptible TB, the cure rate of MDR-TB reaches only 54%, making it known as a fatal disease^2, 3^. Accordingly, identifying the risk factors associated with MDR-TB is of great significance, which may assist in the guidance of intervention measures, promote development of follow-up strategies in specific susceptible populations, and help decision-making in terms of resource allocation.

Several risk factors have been identified for MDR-TB^4^. Among them, previous treatment ranks the strongest and most frequent determinant of MDR-TB, which may be related to the selective pressure of suboptimal regimens or treatment interruptions^4–8^. Other factors include younger age, foreign born, human immunodeficiency virus (HIV) infection, smoking or other substance abuse, being a health care worker and so on^9–11^. However, as the association between most risk factors and MDR-TB differed in different regions and study designs varied, further insight into this area is required.

Recently, along with the convergence of the diabetes mellitus (DM) and TB epidemics, the high prevalence of DM among MDR-TB patients is a serious cause for concern, with a range of 10–23% of MDR-TB patients having DM^12–15^. Whether DM, usually accompanied with altered immunity, has an effect on MDR-TB transmission, as similar with other immunodeficiency related disease (e.g. HIV), is yet to be determined^16^. Findings from studies exploring the associations between DM and MDR-TB have been discordant and some studies did not consider potential confounding factors^17–19^. Thus we conducted a meta-analysis aimed to determine whether DM was an independent risk factor for MDR-TB.

## Methods

### Literature search

This review was registered in the International Prospective Register of Systematic Reviews PROSPERO (registration number: CRD42017057430). Electronic literature search of PubMed, Web of Science and EMBASE databases between the earliest available indexing dates to July 12, 2016 were conducted. The search strategy for PubMed was: (“Diabetes Mellitus”[Mesh] OR “diabetic”[tiab] OR “diabetes”[tiab]) AND “tuberculosis”[Mesh] AND (“Tuberculosis, Multidrug-Resistant”[Mesh] OR “drug resistance”[Mesh] OR “multidrug-resistant”[tiab] OR “multidrug resistant”[tiab] OR “multidrug resistance”[tiab] OR “drug-resistant”[tiab] OR “drug resistant”[tiab]). Web of Science was searched with the search strategy as follows: (“Diabetes Mellitus” OR “Diabetes Mellitus, Type 2” OR “diabetic” OR “diabetes”) AND “tuberculosis” AND (“multidrug-resistant” OR “multidrug resistant” OR “multidrug resistance” OR “drug-resistant” OR “drug resistant” OR “drug resistance”). EMBASE was searched with: (“Diabetes Mellitus”[Emtree] OR “diabetic”[tiab] OR “diabetes”[tiab]) AND “tuberculosis”[Emtree] AND (“drug resistant tuberculosis”[Emtree] OR “drug resistance”[Emtree] OR “multidrug-resistant”[tiab] OR “multidrug resistant”[tiab] OR “multidrug resistance”[tiab] OR “drug-resistant”[tiab] OR “drug resistant”[tiab]). Reference lists of all identified publications were reviewed to identify additional studies. No restrictions were set with respect to language in the entire search process.

### Inclusion and exclusion criteria

Studies were considered eligible for inclusion based on the following criteria: (1) exploring the association between DM and MDR-TB with consideration of potential confounding factors; (2) designed as cohort, case-control, or cross-sectional studies; (3) adjusted odds ratio (OR) and 95% confidence intervals (CIs) were reported or could be calculated. In several studies, the adjusted OR was unavailable in multivariate analysis, due to non-significant statistical results during univariate analysis. Excluding those studies would bias the pooled result towards favoring DM as a risk factor. In these cases, we used a statistically non-significant crude OR instead of the unavailable adjusted OR.We excluded duplicated publications and conference abstracts.

### Data extraction and definitions

Two authors (Qianqian Liu and Wenzhang Li) independently assessed the eligibility of all included studies. Disagreements were resolved by discussion. The following variables were extracted: title, name of first author, publication year, sample size, the country in which the study was conducted, study design, age, gender, MDR-TB type (primary or secondary or any), adjusted OR (or crude OR) and 95% CI, variables adjusted in multivariate regression model. MDR-TB is defined as MTB strains that show resistance at least to rifampicin and isoniazid. Secondary or primary MDR-TB was defined as patients who acquired MDR-TB with or without previously treatment, respectively.

### Quality assessment

We evaluated the methodological quality of all studies in the meta-analysis using the Newcastle-Ottawa scale (NOS), which is composed of the following aspects: selection, comparability, and exposure (case-control or cross-sectional studies) or outcome (cohort studies). The maximum score was nine points. Studies with NOS score <3, 7> NOS score ≥3 and NOS score ≥7 were considered to be of poor, median, high quality, respectively^20^.

### Statistical analysis

The *χ* ^2^ based Q statistics and *I* ^2^ test were used to assess the between-study heterogeneity (significant at *p* < 0.10 and/or *I* ^2^ > 50%). The pooled OR and 95% CIs were calculated using the random effects model. Subgroup analyses were performed by study design, ethnicity and MDR-TB type. Sensitivity analysis was also performed to explore the sources of heterogeneity. Publication bias was assessed by Begg rank correlation and Egger weighted regression test methods^21^. All statistical analyses were conducted by the metan program in STATA version 12.0 (StataCorp, College Station, Texas), using two-sided *p* values.

## Results

### Identification of eligible studies

**Figure 1:**
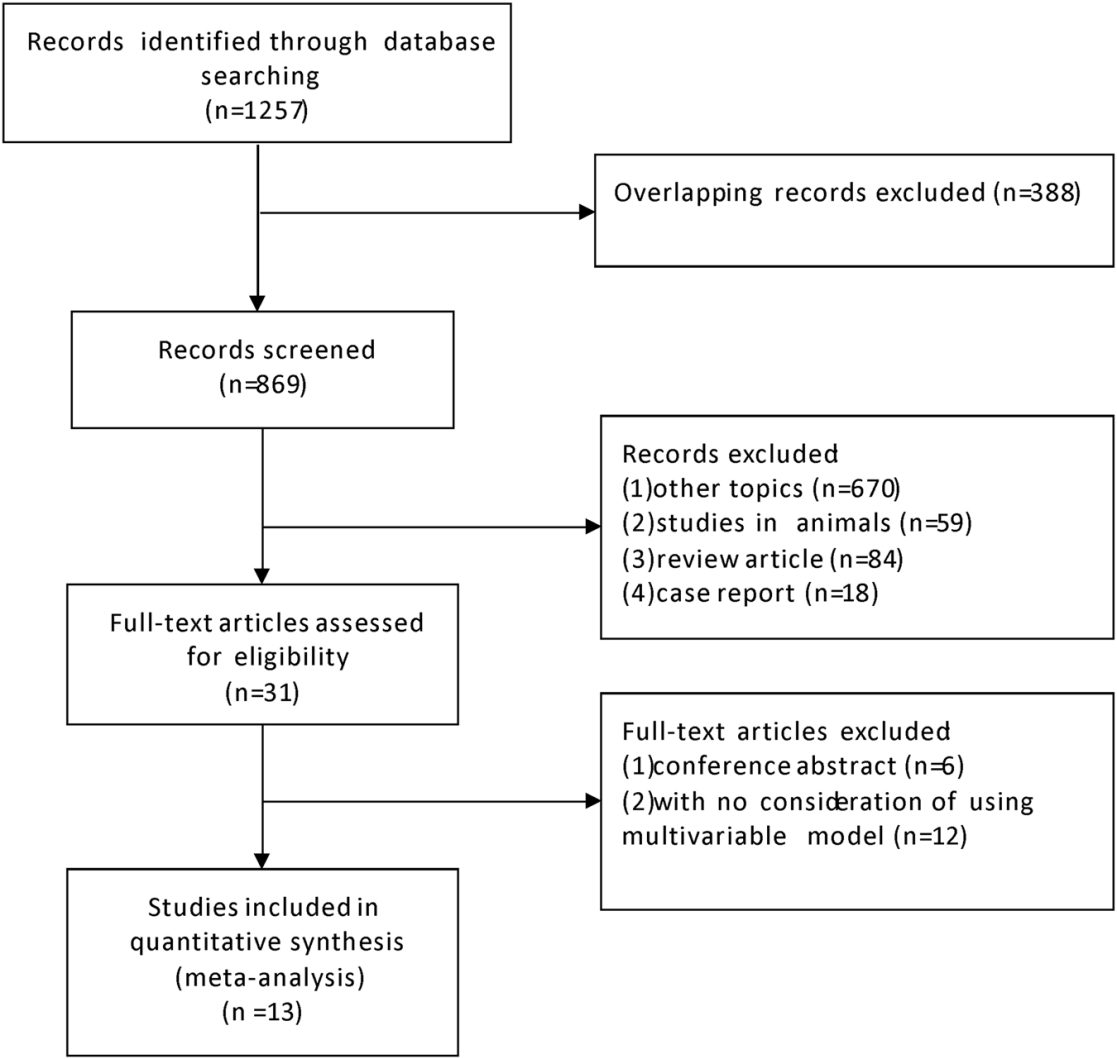
Flow diagram of included studies.

A total of 1257 studies were identified after the initial search (Fig. 1). After excluding over-lapping articles, the remaining 869 studies were screened by reading the titles and abstracts. 670 studies were further excluded for being irrelevant to the topic. We also excluded studies which were conducted in animals, review articles or case reports. Out of 31 studies reviewed in full, six were excluded for being conference abstracts and 12 were excluded because they did not considering using multivariable models to adjust for covariates. We ultimately included 13 studies in this meta-analysis^10, 22–33^.

### Study characteristics

The characteristics of all included studies are presented in Table 1. These 13 studies were published between 2001 and 2015, including a total of 9289 individuals with TB, distributed in Asia (two in China, one in Saudi Arabia, one in Georgia, one in Bangladesh, one in Korea, one in Thailand), the Americas (three in Mexico, two in USA) and Europe (one in Spain). Among those studies, eight were designed as case-control studies, one was a cohort study and four were cross-sectional studies. Four studies clearly stated the MDR-TB type (primary or secondary), while the other nine studies did not classify patients according to history of anti-TB treatment.

**Table 1:** Characteristics of studies included in the meta-analysis.

Author	Year	Country	Sample size	Mean age	Male (%)	Design	MDR-TB type	Crude or Adjusted OR (95% CI)	Variables adjusted in multivariable modela	NOS score
Pérez-Navarro LM	2015	Mexico	409	43	65%	case-control	any	AOR = 3.5 (1.1–11.1)	age	6
Mi F	2014	China	621	NA	NA	cross-sectional	primary and secondary	OR = 1.3 (0.6–2.8) for primary MDR-TB OR = 0.5 (0.2–1.1) for secondary MDR-TB	age, sex, occupation, resident area, previously treated of TB	9
Gómez-Gómez A	2015	Mexico	175	47.5–50.0	70.86%	case-control	any	AOR = 2.51 (1.11–5.67)	age, sex, smoking history, malnutrition, chronic alcohol abuse, and other underlying illnesses	8
Fisher-Hoch SP (1)	2008	USA	1442	NA	486/1442	cross-sectional	any	AOR = 2.14 (1.10–4.17)	age, gender, alcohol, drug abuse, HIV infection and history of previous TB infection	8
Fisher-Hoch SP (2)	2008	Mexico	1436	NA	NA	cross-sectional	any	AOR = 1.80 (1.13–2.87)	age, gender	8
Suárez-García I	2009	Spain	696	NA	67.96%	case-control	any	OR = 1.84 (0.53–6.33)	alcohol abuse, age, previous treatment	8
Hsu AH	2012	China	1008	NA	68.10%	cross-sectional	primary and secondary	AOR = 0.95 (0.34–2.68) for primary MDR-TB AOR = 1.52 (0.59–3.95) for secondary MDR-TB	age, sex	9
Magee MJ	2015	Georgia	318	49	75.20%	cohort	primary	AOR = 2.27 (1.02–5.08)	age, sex, HIV status, smoking status	9
Rifat M	2014	Bangladesh	1000	37	61.0%	case-control	any	AOR = 2.56 (1.51–4.34)	age group, educational status, occupation, smoking status	8
Singla R	2006	Saudi Arabia	692	32.2–48.2	64.60%	cross-sectional	any	OR = 0.3 (0.04–2.37)	age, sex	9
Bashar M	2001	USA	155	NA	84.50%	case-control	any	AOR = 5.3 (1.9–14.7)	HIV and homelessness	6
Min JH	2005	Korea	195	43.8–48.6	77.40%	case-control	primary	AOR = 2.68 (1.05–6.86)	smoking and age	7
Song QS	2015	China	954	45.99–51.34	>70%	case-control	any	AOR = 1.26 (1.11–1.95)	inadequate regimen, insufficient dose, floquirotone, compliance	6
Jitmuang A	2015	Thailand	188	43.87–50.57	52.13%	case-control	any	OR = 1.28 (0.54-3/02)	age, sex, previous TB, HIV, alcohol consumption, positive AFB smear	7

Abbreviations: MDR-TB, multidrug resistant tuberculosis; OR, odds ratio; CI, confidence interval; AOR, adjusted odds ratio; NA, not available; TB, tuberculosis; HIV, human immunodeficiency virus; AFB, acid-fast bacilli.

### Methodological quality

As shown in Table 1, the NOS scores of all included studies ranged from 6 to 9 points, with 12 out of 13 studies deemed to be of high quality. In terms of selection and outcome bias, all studies met all criteria. As for comparability bias, two studies did not adjust for age in their multivariate analyses. In assessing bias related to exposure, five studies obtained the risk factor data according to self report or medical record only, and seven studies did not report the non-response rate in both case and control groups.

### Statistical results

**Figure 2:**
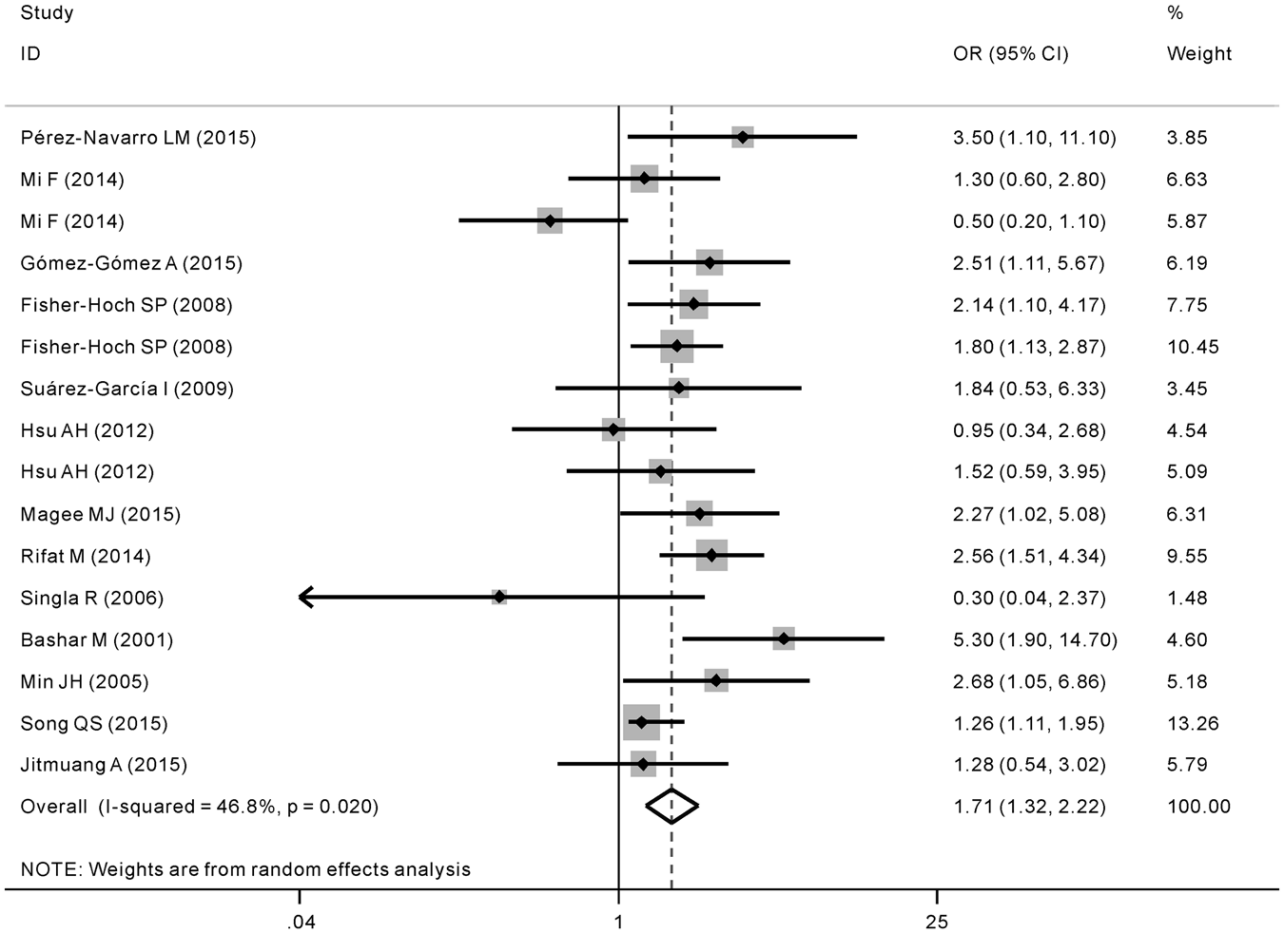
Forest plot of odds ratio (OR) assessing the association between diabetes mellitus and multidrug resistance tuberculosis. CI, confidence interval.

**Figure 3:**
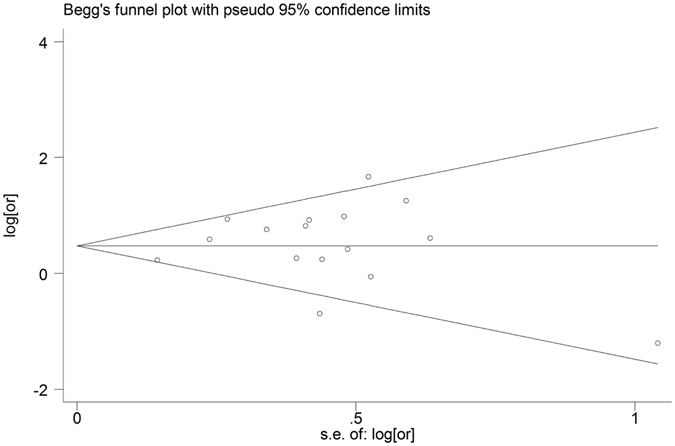
Funnel plots for publication bias.

The overall pooling result of all included studies is shown in Fig. 2 and there was statistical heterogeneity observed among studies (*P* = 0.02, *I* ^2^ = 46.8%). Meta-analysis indicated significant association between DM and MDR-TB, i.e., patients with DM were more likely to have MDR-TB (OR = 1.71; 95% CI = 1.32, 2.22). Sensitivity analysis revealed that most of the heterogeneity derived from one of Mi *et al*. studies, which focused upon the impact of DM as a risk factor on secondary MDR-TB. When we re-analyzed the data after excluding this study, the between-study heterogeneity was significantly reduced (*P* = 0.11, *I* ^*2*^ = 32.3%) and the result still confirmed that DM was an independent risk factor for MDR-TB (OR = 1.83, 95% CI: 1.45–2.31). The symmetric funnel plots shown in Fig. 3 and the lack of significance in the Egger’s test (*P* = 0.561) both indicated that there was no evidence of publication bias.

The subgroup analyses results by study design, MDR-TB type and ethnicity are presented in Table 2. Stratification of the data by study design showed that the OR was 1.25 (95% CI: 0.82–1.91) for cross-sectional studies, and was 2.14 (95% CI: 1.51–3.02) for longitudinal studies. When stratified by MDR-TB type, the pooled OR was 1.69 (95% CI: 1.09–2.62) for primary MDR-TB, 1.94 (95% CI:1.42–2.65) for any MDR-TB, however we did not find that DM was a risk factor for secondary MDR-TB (OR = 0.85, 95% CI: 0.29–2.54). DM was significantly associated with MDR-TB in both Caucasian (OR = 2.26, 95% CI: 1.66–3.07) and Asian (OR = 1.40, 95% CI: 1.01–1.95) subgroups. Compared with the overall pooling analysis, the heterogeneity reduced during subgroup analyses by MDR-TB type, ethnicity, and study design, especially in the Caucasian and primary MDR-TB subgroups.

**Table 2:** Subgroup analyses.

Factors	Subgroups	Number of studies	Pooled OR (95% CI)	*I* ^*2*^	*P*_heterogeneity_
Study design	Cross-sectional	7	1.25 (0.82–1.91)	45.8%	0.09
	Longitudinal	9	2.14 (1.51–3.02)	48.6%	0.05
MDR-TB type	Primary MDR-TB	4	1.69 (1.09–2.62)	2.9%	0.38
	Secondary MDR-TB	2	0.85 (0.29–2.54)	65.7%	0.09
	Any	10	1.94 (1.42–2.65)	48.6%	0.04
Ethnicity	Caucasian	6	2.26 (1.66–3.07)	0.0%	0.50
	Asian	10	1.40 (1.01–1.95)	47.8%	0.05

Abbreviations: OR, odds ratio for MDR-TB due to the presence of DM; CI, confidence interval; MDR-TB, multidrug resistant tuberculosis.

## Discussion

The growing prevalence of TB-DM comorbidity worldwide has provided a new challenge to clinical management and health systems control strategy^34^. It was observed that patients who have DM complicated with TB often experience delayed sputum culture conversion, increased risk of death and recurrence^29, 35–37^. What is more, the emergence of MDR-TB makes the adverse anti-TB treatment outcomes in TB-DM comorbidity even worse, which may increase treatment related economic burden, promote the transmission of MDR-TB, and even accelerate the generation of extensively drug-resistant-TB (XDR-TB). This meta-analysis addressed the association between DM and MDR-TB using 13 selected studies and demonstrated that complicating DM was a significant independent risk factor for MDR-TB, and the risk effect is robust regardless of ethnicity. We didn’t detect any evidence of publication bias. Our data provide a basis for the prioritization of early detection screening measures for MDR-TB among TB patients who are complicated with DM, which may be cost-effective for these patients^38, 39^. In addition, a more intensive anti-TB regimen and careful MDR-TB follow-up might also be suggested in those patients.

The reasons for a higher MDR rate in patients with TB-DM comorbidity are not thoroughly understood, however they may differ by different MDR type (primary or secondary). The combination of impaired immune system in DM and bacterial genetics might be a reasonable explanation for primary MDR. It has been reported that poor glucose control is often associated with dysfunction of phagocytosis, reactive oxygen species (ROS) production, chemotaxis and T-cell reaction in DM patients^40^. On the other hand, MDR strains are shown to be less virulent due to heterogeneous mutations and they are less likely to lead to secondary TB cases compared with drug sensitive strains^41–43^. Then the less fit MDR strains are more likely to flourish in immunocompromised DM patients, which lead to the higher primary MDR-TB in those patients. The situation seems to be more complex with regard to the mechanisms of secondary MDR-TB in DM. Possible explanations include higher mycobacterial burden, altered pharmacokinetics of anti-TB drugs and lower treatment adherence, which promote the selection of MDR strains by anti-TB drugs^40, 44–46^.

Sensitivity analyses showed that most of heterogeneity belonged to one study of Mi *et al*., which focused upon secondary MDR-TB. However, when we conducted the meta-analysis by excluding this research, we also arrived at consistent result. As heterogeneity reduced during subgroup analyses in this meta-analysis, we postulated that MDR-TB type, ethnicity, and perhaps study design may also contribute to heterogeneity. According to our subgroup analysis by MDR type, DM is an independent risk factor for primary MDR-TB with high homogeneity among included studies, while no association between DM and secondary MDR-TB was identified. Similar to our research, a previous meta-analysis also detected significant association between HIV (also being an immune impaired disease) and primary MDR-TB rather than secondary MDR-TB^16^. However, the small sample size of secondary MDR-TB in our meta-analysis might preclude us from drawing a reliable negative conclusion, and it needs to be further explored.

Although most of our included studies scored high quality using the NOS scale, there are still some aspects that need to be improved. Future studies should obtain information about the risk factors (e.g. the diagnosis of DM) according to objective laboratory data rather than basing on self-report or medical record only, which may otherwise lead to misclassification bias. Furthermore, it is necessary to completely report the non-response rate in both case and control groups, so that the existence of non-response bias can be ascertained.

Some limitations of this meta-analysis should be pointed out. First, in this meta-analysis we included data with different study designs, which may lead to heterogeneity of results. However, the similar positive association detected between DM and MDR-TB in the longitudinal study subgroup, which is more liable to prove a cause-and-effect relationship than cross-sectional studies, might strengthen our conclusion. Second, although we have strictly set the inclusion criteria to studies with multivariate analysis to adjust for potential confounding factors, we still cannot adjust the same covariates in all included studies, which may also weaken our conclusion. Third, the control group was from non-MDR TB patients in some studies while from drug susceptible TB patients in others, which may also lead to variability in the strength of association.

In conclusion, the pooling analysis indicated that DM was an independent risk factor for MDR-TB, especially for primary MDR-TB. In patients with DM-TB comorbidity, effective measures need to be implemented to promote early diagnosis of MDR-TB, and followed by intensive treatment and follow-up.
